# Role of Extracellular Vesicles as Potential Diagnostic and/or Therapeutic Biomarkers in Chronic Cardiovascular Diseases

**DOI:** 10.3389/fcell.2022.813885

**Published:** 2022-01-26

**Authors:** Jose Luis Martin-Ventura, Carmen Roncal, Josune Orbe, Luis Miguel Blanco-Colio

**Affiliations:** ^1^ Vascular Research Laboratory, IIS-Fundación Jiménez-Díaz, Madrid, Spain; ^2^ CIBER de Enfermedades Cardiovasculares (CIBERCV), Madrid, Spain; ^3^ Laboratory of Atherothrombosis, Program of Cardiovascular Diseases, Cima Universidad de Navarra, Instituto de Investigación Sanitaria de Navarra, IdiSNA, Pamplona, Spain

**Keywords:** extracelular vesicles, vascular smooth muscle cells, calcification, aneurysm, peripheral arterial disease

## Abstract

Cardiovascular diseases (CVDs) are the first cause of death worldwide. In recent years, there has been great interest in the analysis of extracellular vesicles (EVs), including exosomes and microparticles, as potential mediators of biological communication between circulating cells/plasma and cells of the vasculature. Besides their activity as biological effectors, EVs have been also investigated as circulating/systemic biomarkers in different acute and chronic CVDs. In this review, the role of EVs as potential diagnostic and prognostic biomarkers in chronic cardiovascular diseases, including atherosclerosis (mainly, peripheral arterial disease, PAD), aortic stenosis (AS) and aortic aneurysms (AAs), will be described. Mechanistically, we will analyze the implication of EVs in pathological processes associated to cardiovascular remodeling, with special emphasis in their role in vascular and valvular calcification. Specifically, we will focus on the participation of EVs in calcium accumulation in the pathological vascular wall and aortic valves, involving the phenotypic change of vascular smooth muscle cells (SMCs) or valvular interstitial cells (IC) to osteoblast-like cells. The knowledge of the implication of EVs in the pathogenic mechanisms of cardiovascular remodeling is still to be completely deciphered but there are promising results supporting their potential translational application to the diagnosis and therapy of different CVDs.

## Cardiovascular Diseases

CVDs, including heart, coronary, cerebrovascular, peripheral and aortic diseases, are the leading cause of morbidity and mortality in developed countries (Mannello and Medda, 2012). Atherosclerosis, considered the major precursor of CVDs, is a chronic pathology affecting large and medium size arteries that begins early in life, and progresses silently from its subclinical form to clinical symptoms according to the exposure to environmental risk factors (cholesterol, diabetes, hypertension, smoking, stress, sedentarism, microbioma, etc), and non-modifiable determinants such as age (Bray et al., 2021; Libby, 2021). Despite the therapeutic advances in controlling traditional risk factors, and the irruption of novel approaches including targeting inflammation, CVDs remain the primary cause of mortality worldwide, accounting for almost a third of annual deaths, 17.3 million per year, that are expected to grow to more than 23.6 million by 2030 due to lifestyle changes and aging (Roth et al., 2017). Arterial alterations can go unnoticed until symptoms develop, a sign of advanced disease, and thus are associated to a high risk of ischemic complications and death (Acosta et al., 2006; Roth et al., 2017; Timmis et al., 2020). In this context, extracellular vesicles (EVs) emerge as new players in the crosstalk between vascular and circulating cells, participating in cell to cell communication processes, and being biomarkers of cellular activation (Méndez-barbero et al., 2021).

### Peripheral Arterial Disease

**FIGURE 1:**
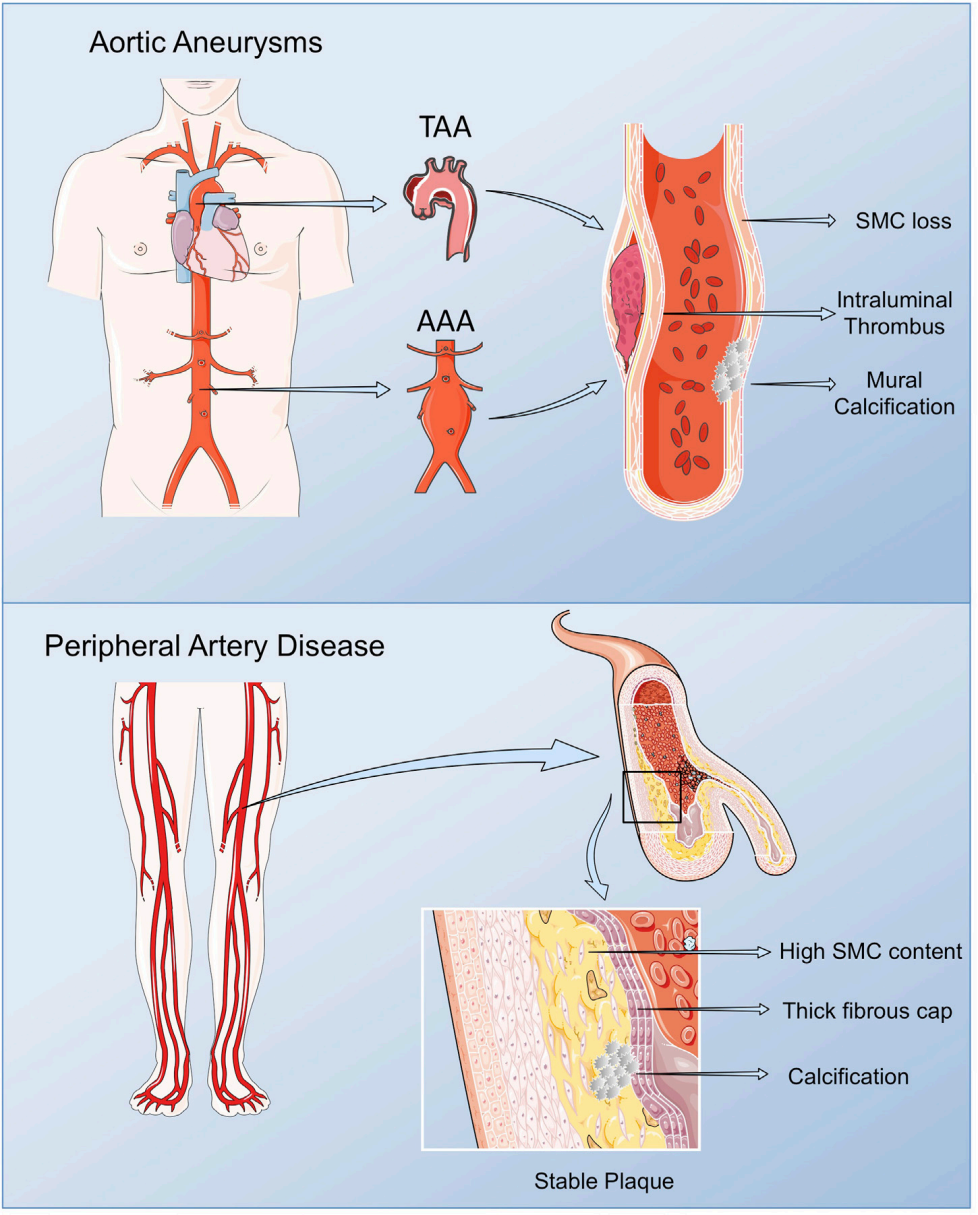
Pathological vascular remodeling. Arterial remodeling in peripheral arteries and thoracic and abdominal aortas present some phenotypic particularities compared with vascular lesions in other vascular beds. Lower limb lesions are most frequently stable with low macrophage content, and a prevalence of vascular SMCs, resulting in plaques rich in collagen and elastic fibers, mostly calcified, with a non-significant lipid core. AAAs are characterized by the presence of an Intra Luminal Thrombus (ILT), rich in red blood cells (RBCs), neutrophils and platelets, and an aortic wall with low number of vascular SMCs in the remaining media, along with immune cell infiltrates, fibroblasts and neovessels in the adventitia. TAA display extensive remodeling of the ECM, vascular SMCs dysfunction and calcification, but ILT is not present. Figure created using Servier Medical Art images (https://smart.servier.com).

PAD includes a range of non-coronary arterial syndromes caused by alterations in the structure and function of arteries other than those supplying the heart or the brain, although in the current review, we will focus on lower extremity PAD referring to the chronic lower limb ischemia of atherosclerotic origin affecting the femoral, popliteal and saphenous arteries (Gerhard-Herman et al., 2017; Frank et al., 2019). PAD affects around 200 million people worldwide, and increases with age, presenting a prevalence of 10–25% in people older than 55 years, rising up to 40% on those older than 80. PAD is associated to a diminished quality of life affecting mobility, and in its more severe form, chronic limb threatening ischemia (CLTI), it might lead to limb amputation, and mortality in high rates (Fowkes et al., 2017). Taking in consideration that PAD is frequently accompanied by atherosclerosis in other vascular beds, it presents a superior risk of ischemic events and death compared with other CV pathologies (Fowkes et al., 2013; Jirak et al., 2018). As such, when associated with other comorbidities, specifically diabetes, the gold standard for PAD diagnosis, the ankle brachial index, losses sensitivity due to arterial calcification, contributing to its silent rates (Hajibandeh et al., 2017; Jirak et al., 2018). In consequence, and despite its bad prognosis, PAD still remains greatly underdiagnosed and undertreated (Norgren et al., 2007). The morphological analysis of femoral plaques revealed some differences when compared with those in coronary and cerebral arteries. In lower limb lesions, macrophage content is lower, while vascular SMCs are predominant, originating plaques rich in collagen and elastic fibers, mostly calcified, with a non-significant lipid core (Derksen et al., 2010; Kamenskiy et al., 2018). This phenotype stabilizes the plaque, slows its progression, and enables the activation of compensatory mechanisms, including collateral circulation, in surrounding tissues (Poredoš et al., 2021) (Figure 1).

### Aortic Valve Stenosis

Degenerative AS is the most frequent form of acquired valvular heart disease in the developed countries, and the second most frequent cause for cardiac surgery, which prevalence will further increase with the aging of the population. The prevalence of AS in patients older than 75 years of age is 12.4%, and 3.4% have severe AS with an associated risk of death of 50% at 2 years (Iung and Vahanian, 2006; Nkomo et al., 2006; Kanwar et al., 2018). Normal aortic valves are composed of valvular endothelial cells (ECs), the ICs and extracellular matrix (ECM), while pathological aortic valves are characterized by endothelial dysfunction, lipoprotein accumulation, chronic inflammation, and calcium nodule deposits. Consequently, these pathological changes lead to the thickening and stiffness of the valve leaflets, restricting their opening and imposing an increased afterload on the left ventricle (O’Brien, 2006; Sritharen et al., 2017). Despite the associated clinical consequences, there is currently no effective therapy for AS other than aortic valve replacement (Aymond et al., 2021; Mantovani et al., 2021).

### Aortic Aneurysms

AAs occurs when the progressive weakening of the aortic wall causes the aorta to enlarge and exceeds more than 3 cm. AAs are usually asymptomatic and are often detected as an incidental finding during the investigation of an unrelated problem or as a consequence of radiological screening. The only way to prevent aortic rupture or disection in patients with an AAs > 5–5.5 cm is surgery (Klink et al., 2011; Milewicz and Ramirez, 2019). AAs can be distinguished by their etiology into degenerative aneurysms and those associated with hereditary disorders or by their different location into abdominal aortic aneurysm (AAA) or thoracic aortic aneurysm (TAA) (Sakalihasan et al., 2005; Pinard et al., 2019). AAA is a major health problem, causing about 1–2% of male deaths in economically developed societies. TAA is a relatively common condition, found in up to 8% of men aged >65 years, and is responsible for considerable cardiovascular morbidity and mortality.

AAA share main CV risk factors with atherosclerosis, while the major risk factors for TAA are hypertension and an underlying genetic alteration or the presence of a bicuspid aortic valve (BAV). AAA is characterized by the presence of an Intra Luminal Thrombus (ILT), mainly composed by red blood cells (RBCs), neutrophils and platelets and an aortic wall with reduced number of vascular SMCs in the remaining media, along with immune cell infiltrates, fibroblasts and neovessels in the adventitia (Michel et al., 2011). Despite the origin of AAA still being poorly understood, proteolysis, oxidative stress, vascular SMCs phenotypic switch and apoptosis, immune-inflammatory responses and neoangiogenesis are mechanisms implicated in the formation and progression of AAA (Torres-Fonseca et al., 2019). Although AAA and TAA share some common mechanisms including proteolytic elastic tissue degeneration and vascular SMCs dysfunction, they present some striking differences: presence (AAA) or abscence (TAA) of ILT; secondary (AAA) versus primary (TAA) pathology of vascular SMCs; linkage (AAA) or not (TAA) to atheroma; monogenic (TAA) versus polygenic (AAA) determinants; age and gender issues (Michel et al., 2018; Martin-Blazquez et al., 2021) (Figure 1).

## Extracellular Vesicles

**FIGURE 2:**
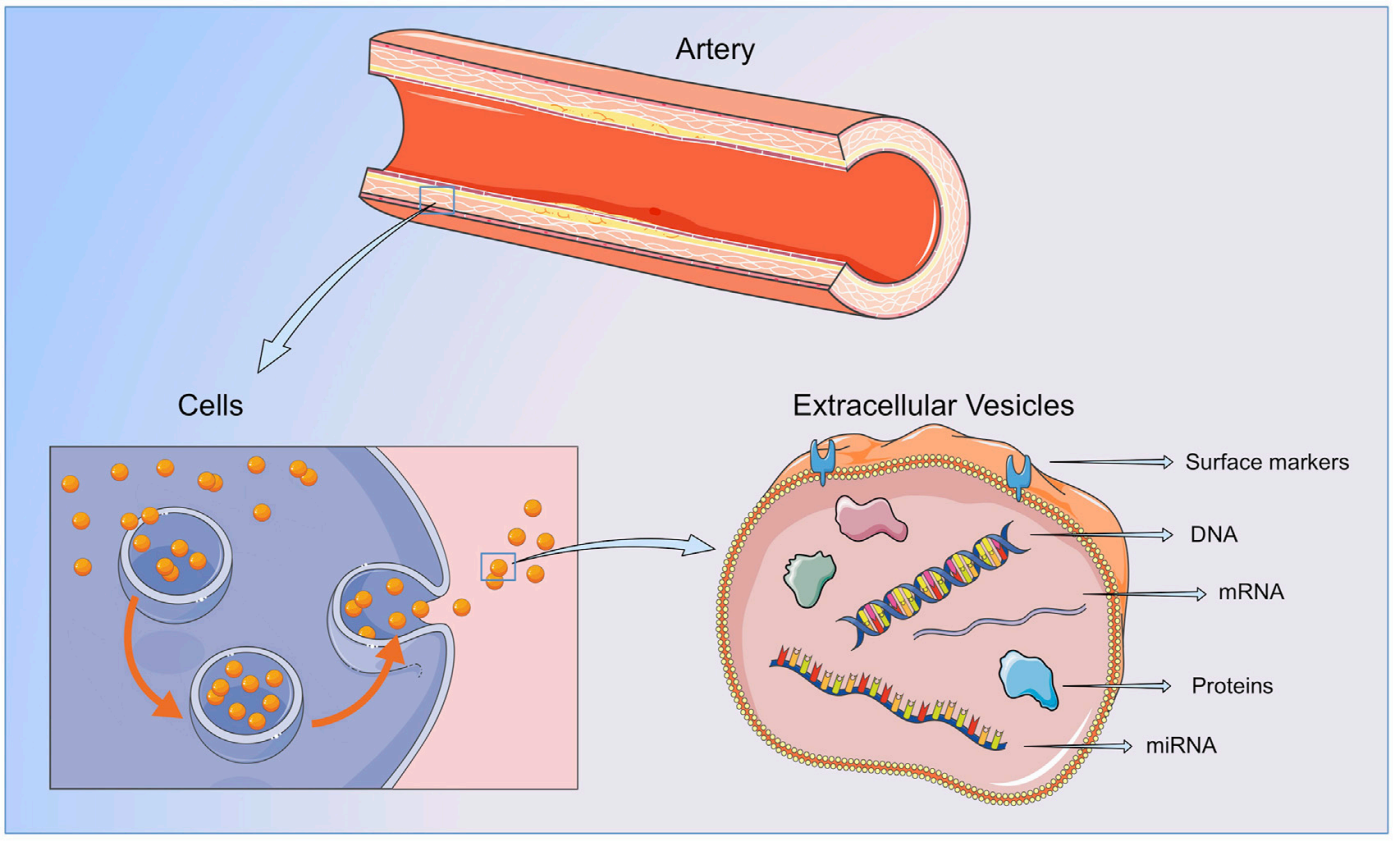
Extracellular vesicles. EVs contain lipids, protein and nucleic acids from the cell or organ of origin and are released to the bloodstream by all cellular components of the vasculature and blood. By transferring their content to neighboring or distant cells or by direct interaction with the ECMs components, EVs are able to participate in all the steps of vessel remodeling. Figure created using Servier Medical Art images (https://smart.servier.com).

EVs are spherical lipid bilayers without nucleus, released to the extracellular space by most cell types, containing lipids, proteins, metabolites and nucleic acids from the cell of origin. The lipid bilayer protects EVs content from the activity of endogenous DNases, RNases or proteinases, and temperature or pH changes, and enables their separation from all body fluids and cell culture medium (Momen-Heravi et al., 2018) (Figure 2).

EVs are produced and released to the bloodstream in physiological and pathological conditions by all vascular and circulating cells, displaying a wide range of functions in the vasculature and in target tissues (Giró et al., 2021). The biological activities of EVs in recipient cells are displayed through different mechanisms (Russell et al., 2019). For instance, they can participate in receptor-ligand interactions promoting the activation of signaling pathways in host cells, release their content to the cytoplasm of recipient cells by fusion with the plasma membrane, or be internalized through endocytosis, phagocytosis or micropinocytosis. Finally, by carrying bioactive molecules in their surface, EVs can directly modify target proteins or ECMs components (van Niel et al., 2018). In this way, EVs regulate vascular homeostasis and participate in a myriad of pathological processes, including atherosclerosis initiation and progression, and aortic wall dilation (Méndez-barbero et al., 2021; Saenz-pipaon et al., 2021). Moreover, being their cargo particular to the cell type and the stimuli triggering their release, the analysis of their content might be useful to understand the pathophysiological condition on which they have been originated (Shah et al., 2018; Théry et al., 2018). In addition, EVs can lead to the identification of novel diagnostic and prognostic biomarkers and therapeutic targets.

Traditionally, EVs have been classified according to their size and biogenesis in: exosomes, with diameters from 30 to 150 nm, generated by the inward budding of the endosomal membrane (Colombo et al., 2014); microvesicles, directly shed from the plasma membrane and polydisperse in size (100–1000 nm of diameter) (Connor et al., 2010; Boulanger et al., 2017; Taylor et al., 2020) and apoptotic bodies, generated in the late steps of programmed cell death with diameters of 1000–5,000 nm (Théry et al., 2018; van Niel et al., 2018). However, due to the significant overlap in size and composition between exosomes and microvesicles and following the latter recommendations (Théry et al., 2018), in this review we will use the umbrella term EVs to refer to exosomes and microvesicles (Théry et al., 2018; Russell et al., 2019).

## Extracellular Vesicles and Cardiovascular Diseases

EVs have attracted considerable interest in the CV field as reservoirs of molecules produced during arterial remodelling, and/or after acute or chronic ischemic events (Giró et al., 2021), and thus have been proposed as biomarkers in different CV conditions (Table 1). Likewise, increased levels of total EVs or specific EVs subpopulation, for instance those derived from platelet, ECs, erythrocytes or leukocytes, have been associated with the presence of CV risk factors (Amabile et al., 2014), including diabetes (Li et al., 2016), hypertension (Preston et al., 2003), hypercholesterolemia (Amabile et al., 2014) and smoking (Gordon et al., 2011), and with subclinical and clinical atherosclerosis (Suades et al., 2015; Saenz-Pipaon et al., 2020). In coronary pathologies, circulating EVs have been correlated with inflammatory and thrombophilic conditions. As such the number of endothelial and platelet derived EVs have been correlated with the circulating levels of IL-6 and CRP in patients with coronary heart disease (Cui et al., 2013); moreover *in vitro*, medium size vesicles from acute coronary syndrome patients displayed procoagulant activity, which was prevented when phosphatidylserine was blocked with lactadherin (Liu et al., 2016). EVs have been also postulated as markers of carotid plaque instability, reporting increased levels of platelet, endothelial, leukocyte and erythrocyte EVs in patients with myocardial infarction when compared with subjects with unstable and stable angina (Cui et al., 2013; Wekesa et al., 2014; Schiro et al., 2015; Liu et al., 2016; Suades et al., 2016; Vagida et al., 2016). Similar results were obtained when assessing the number of tissue factor and annexin V positive procoagulant EVs subpopulations (Cui et al., 2013; Wekesa et al., 2014). Coronary patients also displayed increased levels of endothelial EVs according to worse vascular function (Amabile et al., 2005; Koga et al., 2005; Werner et al., 2006), and an association between elevated endothelial and erythrocyte EVs and CV events has been also reported (Sinning et al., 2011; Fan et al., 2014; Giannopoulos et al., 2014). Furthermore, in stroke patients elevated levels of specific subpopulations of endothelial derived EVs were associated to worse outcome (Simak et al., 2006; Jung et al., 2009; Li and Qin, 2015). In PAD, circulating EVs are mainly considered platelet activation markers (Zeiger et al., 2000; Saenz-Pipaon et al., 2020), while in AAA, EVs were enriched in proteins involved in the main pathological mechanisms leading to AAA development and progression, including oxidative stress, inflammation and thrombosis (Martinez-Pinna et al., 2014). Besides their role as biomarkers, EVs display biological activities and induce cellular responses *in vitro* and *in vivo* also during vessel remodelling, modulating endothelial (dys)function, leukocyte recruitment, foam cell formation, VSMCs proliferation and migration, apoptosis and necrotic core formation, plaque rupture and thrombosis. This topic has been extensively addressed in recent reviews (Zarà et al., 2019; Giró et al., 2021; Patel et al., 2021).

**TABLE 1:** Summary of studies showing a correlation between the levels of total or specific cell type EVs subpopulations, measured by flow cytometry, in circulation (plasma) and cardiovascular diseases.

Disease/patient group (*n* = number)	EVs type	Outcome	References
844 individuals of the Framingham Offspring cohort (mean age 66 + 9 years, 57% women) without cardiovascular disease	Endothelial (e)EVs subsets	Association of eEVs with hypertension, triglycerides and metabolic syndrome and the Framingham risk score	Amabile et al. (2014)
	CD144+		
	CD31^+^CD41^−^		
Systemic review and meta-analysis of 48 studies involving 2,460 patients with type 2 DM and 1,880 non-diabetic controls	Specific surface markers	Total EVs, pEVs, mEVs and eEVs were higher in type 2 DM vs. controls	(Li et al., 2016)
Three groups: 1) untreated patients with severe uncontrolled hypertension, n = 24; 2) untreated patients with established mild hypertension, *n* = 19; and 3) normotensive volunteer subjects *n* = 16	eEVs: CD31^+^CD42^−^	eEVs and pEVs were significantly increased in the severely hypertensive group	Preston et al. (2003)
	Platelet (p)EVs: CD41^+^		
Two groups: 1) High cardiovascular risk patients (*n* = 37); 2) age, gender, and treatment-matched controls (*n* = 37)	Total EVs (Annexin V+)	Total EVs, pEVs, mEVs, and TF + EVs were significantly elevated in high risk patients vs. controls	Suades et al. (2015)
	pEVs		
	TSP1+		
	PAC+		
	PAC + CD62P+	Levels of TF + mEVs and pEVs were associated to atherosclerotic burden	
	Tissue factor (TF)+ pEVs: CD142 + TSP1+		
	TF + monocyte (m)EVs: CD142 + CD14^+^		
Four groups: 40 patients with myocardial infarction (MI), 30 unstable angina (UA), 20 stable angina (SA), and 20 healthy individuals	pEVs: CD41^+^	eEVs and pEVs were significantly elevated in MI and UA vs SA and control	Cui et al. (2013)
	mEVs: CD14^+^	No differences were observed in mEVs and lEVs among the groups	
	Lymphocytes (l)EVs: CD4^+^		
	eEVs: CD144+	TF + EVs were higher in MI and UA	
	TF + EVs: CD142+	eEVs and pEVs levels correlated with IL-6 or CRP in coronary heart disease patients	
Patients with newly diagnosed acute coronary syndrome (ACS) were divided into 3 groups: 1) UA (*n* = 36), 2) NSTEMI (*n* = 22), and 3) STEMI (*n* = 42); and an additional group of *n* = 10 age and sex matched controls	Total EVs: Lactadherin+	Total EVs, pEVs and eEVs were higher in ACS groups vs controls	Liu et al. (2016)
	pEVs: CD41a+		
	eEVs: CD31^+^	Leu- and eryEVs were higher in the STEMI group vs UA and NSTEMI groups (both *p* < 0.05)	
	mEVs: CD14^+^		
	B-cells EVs: CD19^+^	*In vitro*, EVs form ACS patients displayed procoagulant activity	
	T cells EVs: CD3^+^		
	Erythrocyte (ery)EVs: CD235a + TF + EVs: CD142+		
CAD patients undergoing endarterectomy (*n* = 42), and age- and sex-matched controls (*n* = 73)	Ann V + EVs	Annexin V + EVs and pEVs subsets, were higher in cases vs. controls	Wekesa et al. (2014)
	pEVs subsets		
	CD41^+^		
	Ann V + CD41^+^	eEVs subsets were higher in patients with unstable vs. stable plaques	
	eEVs subsets		
	CD31^+^CD41^−^		
	CD144+	eEVs and pEVs were significantly higher in patients with carotid stenosis vs. controls	
	CD146+		
	CD105+		
Patients undergoing carotid endarterectomy: *n* = 19 asymptomatic and *n* = 51 symptomatic patients (*n* = 51); and *n* = 20 healthy age-matched controls	eEVs: Ann V + CD31^+^CD42b−		Schiro et al. (2015)
	pEVs: Ann V + CD31^+^CD42b+	No differences were observed between asymptomatic vs. symptomatic patients
STEMI patients (*n* = 40) treated by percutaneous coronary intervention (PCI); age, gender, risk factors and pharmacological treatments matched control group (*n* = 20); and patients recovering from STEMI (*n* = 20)	Total EVs (Ann V+)	STEMI patients present increased levels of total EVs, LeuEVs subsets, eEVs subsets and PEVs	Suades et al. (2016)
	eEVs		
	CD31^+^		
	CD146+		
	CD62E+		
	pEVs: CD61^+^		
	Leukocytes (leu)EVs: CD45^+^		
	lEVs: CD45+/CD3+		
	mEVs: CD14^+^		
	neutrophil EVs: CD66b+		
	TF + EVs: CD142+		
17 healthy volunteers and 13 ACS.	Magnetic nanoparticles conjugated with anti-CD63/CD31 or anti-CD31 for eEVs, or with anti- CD63/CD41a or anti-CD41a antibodies for pEVs	ACs patients presented increased levels of EVs, mainly of platelet origin	Vagida et al. (2016)
44 end-stage renal failure patients (ESRF), and 32 healthy subjects	Ann V + EVs	Annexin V + EVs, eEVs, pEVs and eryEVs were increased in ESRF patients vs. controls	Amabile et al. (2005)
	eEVs subsets		
	CD31^+^		
	CD144+		
	pEVs: CD41^+^		
	eryEVs: CD235a+	Only eEVs correlated with arterial dysfunction	
	Lymphocyte EVs: CD3^+^		
	myeloid EVs: CD11b+		
	LeuEVs: CD45^+^		
	Neutrophil EVs: CD66b+		
232 patients with DM and 102 controls	eEVs: CD144 + CD42b−	eEVs levels were increased in DM vs. control	Koga et al. (2005)
	In DM patients, eEVs were associated to a higher risk for CAD		
CAD patients (*n* = 50)	eEVs: CD31 + Ann V+	Increased eEVs correlated with worse endothelial-dependent vasodilatation and independently predicted severe endothelial dysfunction	Werner et al. (2006)
CAD patients (*n* = 200)	eEVs: CD31 + Ann V+	eEVs were increased in patients with a first major adverse cardiovascular and cerebral events (MACCE)	Sinning et al. (2011)
		In the follow up eEVs were independently associated to higher risk of CV death, need for revascularization or MACCE.	
Healthy controls (*n* = 80), chest paint patients: non-CAD (n = 94), SA (*n* = 111), and ACS (*n* = 145)	eEVs: CD146+	The levels of eEVs were increased in ACS > SA > non-CAD > controls	Fan et al. (2014)
		eEVs levels were associated to higher risk of MACE in ACS group	
STEMI patients (*n* = 51) and age-matched controls (*n* = 50)	Ann V + EVs	eryEVs were increased in STEMI patients vs. controls	Giannopoulos et al. (2014)
	pEVs: CD41^+^	No differences were found in pEvs	
	eryEVs: CD235a+	eryEVs levels were independently associated to a higher risk of MACE during the follow-up	
Stroke patients: 1) mild stroke, *n* = 20; 2) moderate–severe stroke, *n* = 21; 3) age-matched controls, *n* = 23	eEVs subsets	PS + eEVs were increased in stroke patients vs. controls	Simak et al. (2006)
	CD105 + CD41a-CD45− (E + eEVs)	All eEVs subsets were elevated in moderate–severe stroke patients vs. controls	
	CD105 + CD144+ (C + eEVs)		
	CD105 + PS + CD41a− (PS + eEVs)	Brain lesion volume was correlated E + eEVs, PS + eEVs and I+ eEVs levels	
	CD105 + CD54^+^CD45− (I + eEVs)		
Patients with acute stroke (*n* = 73), and patients with vascular risk factors but no stroke events (*n* = 275)	eEVs subsets	Levels of CD31+/AnnV+ and CD62E + eEVs subsets were greater in acute stroke patients vs. controls	Jung et al. (2009)
	CD31+/CD42b-	CD62E + eEVs were strongly associated with stroke severity and infarct volume	
	CD31+/AV+		
		CD62E+	
Patients with acute ischemic stroke (*n* = 68), and age- and sex-matched controls (*n* = 61)	eEVs subsets	CD144+/CD41a−, CD31^+^CD41a−, CD62E+, and Annexin V + CD62E + eEVs, were significantly increased in acute ischemic stroke patients vs. controls	Li and Qin (2015)
	CD144 + CD41a−		
	CD31^+^CD41a−		
	CD62E+	CD144+/CD41a− eEVs were correlated with stroke severity	
	Ann V + CD62E+		
		pEVs: CD41a + CD144−	
18 PAD patients and 12 asymptomatic controls	Total EVs: Lactadherin+	PAD patients presented increased levels of eEVs carrying the monomeric form of C-reactive protein (mCRP)	Crawford et al. (2016)
	pEVs: CD41a+		
	eEVs subsets		
	CD31^+^		
	CD144 +		
	LeuEVs: CD45^+^	Control subjects on statins presented a reduction in mCRP + eEVs	
	mEVs: CD14^+^		
	B-cells EVs: CD19^+^		
	T cells EVs: CD3^+^		
	Neutrophil EVs: CD66b+		
		eryEVs: CD235a+	
		Monomeric (m) or pentameric (p) CRP + EVs	
PAD patients (*n* = 50) and controls (*n* = 50)	eEVs: CD144+	PAD patients present increased levels of shh+ in all EVs subpopulations	Giarretta et al. (2018)
	pEVs: CD42b+		
	LeuEVs: CD45^+^	Shh + eEVs levels correlated with the number of collateral vessels in ischemic thighs of PAD patients (*n* = 18)	
	eryEVs: CD235+		
	Sonic Hedgedog (Shh)+EVs		
PAD patients (*n* = 50) and controls (*n* = 50)	pEVs: CD41^+^	Increased levels of pEVs in PAD patients vs controls	Zeiger et al. (2000)
PAD patients, *n* = 23 with severe disease (critical limb ischemia, CLI), 36 with moderate disease (intermittent claudication, IC), and *n* = 30 healthy controls	pEVs: CD61^+^CD42b+	Gradual increased in pEVs levels according ro severity (CLI > IC > controls)	Tan et al. (2005)
Patients presenting stable angina (*n* = 10), peripheral arterial disease (*n* = 10), NSTEMI (*n* = 11) and STEMI myocardial infarction (*n* = 10), age- and sex matched older controls *n* = 10 and young healthy individuals (*n* = 10)	pEVs subsets	96% of the detected EVs were from platelet origin	van der Zee et al. (2006)
	Ann V + CD61^+^CD62P+		
	Ann V + CD61^+^CD63^+^	CD62P + pEVs increased in patients with NSTEMI and STEMI vs. older controls	
	eEVs: CD62E		
	EryEVs: CD235a		
	T- cells EVs	CD63+pEVs- were increased in patients with PAD, NSTEMI, and STEMI vs. older controls	
	CD4 +		
	CD8^+^		
	mEVs: CD14^+^		
	B cells EVs: CD20^+^		
	Neutrophil EVs: CD66e+		
PAD patients (*n* = 45)	AnnexinV eEVs: CD62E+ pEVs: CD41/61+ LeuEVs: CD11b+ eryEVs: CD235a+	In plasma of PAD patients pEVs were the most abundant subpopulation, followed by eryEVs, eEVs and LeuEVs	Saenz-Pipaon et al. (2020)
	More than 85% of pEVs and eryEVs were Ann V+, while the percentage was lower for eEVs (70%) and LeuEVs (40%)		
	The number pEVs were inversely correlated with procoagulant activity of plasma		
14 PAD patients and 15 normal controls PAD patients were treated with cilostazol (2 weeks) or cilostazol with dipyridamole (14 weeks)	pEVs: CD42^+^	PAD patients presented increased levels of pEVs	Nomura et al. (2004)
		Cilostazol, and further, cilostazol with dipyridamole decreased pEVs levels in PAD patients	
PAD patients (*n* = 19) randomly assigned to Atorvastatin or placebo treatment for 8 weeks	Total EVs: lactadherin	Atorvastatin treatment reduced the number of CD142+, CD62P+ and CD61^+^ pEVs vs placebo treated PAD patients	Mobarrez et al. (2011)
	pEVs		
	CD42a + CD142+		
	CD42a + CD62P+		
	CD42a + CD61^+^		
PAD patients (*n* = 19) randomly assigned to Atorvastatin or placebo treatment for 8 weeks	Total EVs	Both CD144 + eEVs and CD144 + CD142+ eEVs were increased in patients on atorvastatin vs. placebo	Mobarrez et al. (2012)
	eEVs		
	Lactdherin + CD144+		
	Lactadherin + CD144 + CD142+		
22 patients with severe aortic stenosis (AS) and 18 controls	eEVs: CD62E+	pEVs, LeuEVs and eEVs were increased in AVS patients vs. control	Diehl et al. (2008)
	pEVs		
	CD31^+^ CD61^+^	pEVs levels were correlated with shear stress and eEVs with the number of blood monocytes	
	CD62P+		
	LeuEVs: CD11b+		
Patients with severe AS. *n* = 28 with low coronary calcification (CAC) score, and *n* = 27 with high CAC score	eEVs	The levels of pEVs and CD62E + eEVs were increased in high CAC score patients vs. low CAC score group, and correlated to the calcium score	Horn et al. (2016)
	CD144+		
	CD62E+		
	CD31^+^CD41^−^	EVs thrombin generation activity was higher in patients with high CAC score	
	pEVs: CD41^+^		
	EVs trombin generation activity		
56 severe AS patients undergoing transcatheter aortic valve implantation (TAVI)	eEVs	All eEVs subpopulations decreased 3 months after TAVI, along with an increase in the endothelial function	Horn et al. (2015)
	CD144+		
	CD62E+		
	CD31^+^CD41^−^		
	pEVs: CD41^+^		
92 severe AS patients undergoing TAVI	eEVs	The levels of CD62E + eEVs decreased gradually from pre-TAVI to post-TAVI (1 week, 1, 3 and 6 months) determination	Jung et al. (2017)
	CD31 + Annexin+		
	CD31 + Annexin−		
	CD31^+^CD42b−	In contrast, circulating PEVs increased gradually after TAVI	
	CD62E+		
	pEVs: CD31^+^CD42b+		
Patients with severe AS selected for percutaneous replacement of the aortic valve (*n* = 9)	eEVS: CD31 + Ann V+	No differences were observed between pre- and post-operative (5 days) levels of eEVs, pEVs or LeuEVs	Marchini et al. (2016)
	pEVs: CD41 + Ann V+		
	LeuEVs: CD45 + Ann V+		
135 patients undergoing surgical aortic valve replacement	small (s)EVs were quantified by nanoparticle tracking analysis (NTA)	sEVs decreased 24 h post-surgery, and recovered to pre-operative levels 7 days and 3 months post-procedure	Weber et al. (2020)
		No association between sEVs and echocardiographic parameters before or after surgery (7 days and 3 months) were observed sEVs levels were correlated to prosthesis patients mismatch parameters at month 3 post-surgery	
AAA patients (blood samples and mural thrombi, *n* = 20), and sex and age-matched healthy individuals (blood samples, *n* = 20)	Annexin V + EVs	Circulating total EVs were significantly increased in AAA patients vs. controls	Touat et al. (2006)
	pEVs: CD41		
	neutrophil EVs CD15		
	mEVs: CD14	Locally, luminal thrombus layers released larger quantities of annexin V-positive EV, mainly of platelet and neutrophil origin, compared to the intermediate and abluminal layers	
	eEVs: CD106		
	eryEVs: CD235		
Controls (*n* = 66) and thoracic AA (TAA) patients associated to bicuspid aortic valves (BAV) (*n* = 15), or other origins (degenerative, *n* = 23)	Ann V + EVs	The levels of EVs and pEVs were higher in TAA groups vs. control	Touat et al. (2008)
	pEVs: Ann V + CD41^+^		

AAA: abdominal aortic aneurysm; ACS: acute coronary syndrome; AS: aortic stenosis; TAA: Thoracic aortic aneurysm; TAVI: transcatheter aortic valve implantation; CAD: coronary artery disease; CAC: coronary calcification score; DM: diabetes mellitus; ESRF: end-stage renal failure; CLI: critical limb ischemia; IC: Intermittent claudication; MI: myocardial infarction; PAD: peripheral arterial disease; SA: stable angina; UA: unstable angina; Ann V: Annexin V; EVs: Extracellular vesicles; eEVs: endothelial EVs; eryEVs: erythrocyte EVs; LeuEVs: leukocyte EVs; lEVs: Lymphocyte EVs; mEVs: monocyte EVs; pEVs: platelet EVs; (N)STMI: (non) ST Segment Elevation Myocardial Infarction; TF: Tissue factor; TSP-1: Thrombospondin-1; T; PAC: activated αIIbβ3-integrin.

### Extracellular Vesicles: Potential Biomarkers and Biological Effectors in Peripheral Arterial Disease

EVs have been investigated as biomarkers and biological effectors in PAD, displaying different effects according to their cargo and origin. It has been proposed that the elevated levels of endothelial EVs co-expressing the monomeric form of C-reactive protein (CRP) might potentially contribute to inflammation in PAD (Crawford et al., 2016), while endothelial cell derived EVs rich in the pro-angiogenic Sonic hedgehog protein correlated with the number of collateral vessels in ischemic thighs of PAD patients, suggesting their possible role in neovascularization (Giarretta et al., 2018). Among the different EVs lineages, platelet EVs, seemed to be most abundant in plasma of PAD patients (Saenz-Pipaon et al., 2020), and their elevated numbers were correlated with PAD diagnosis and prognosis (Zeiger et al., 2000; Tan et al., 2005; van der Zee et al., 2006). It should be considered, however, that within total platelet EVs, only those co-exposing either P-selectin or CD63 might reflect the degree of platelet activation *in vitro* (van der Zee et al., 2006). Based on those data, it has been suggested the potential benefit of modifying total EVs numbers or subpopulations by pharmacological treatments (Rosińska et al., 2017). As such, cilostazol induced a reduction in the total number of platelet EVs in PAD patients (Nomura et al., 2004), while atorvastatin modified specific platelet EVs subpopulations, reducing those positive for P-selectin, tissue factor and glycoprotein-IIIa (Mobarrez et al., 2011). Interestingly, atorvastatin displayed the opposite effect on endothelial EVs inducing their increase in circulation (Mobarrez et al., 2012).

EVs also encapsulate nucleic acids, mainly mRNA and non-coding RNAs, from the cells or organs of origin, that can be released to the circulation and display biological responses in neighboring or distant cells. As such, small EVs from PAD patients enriched in miR-21, miR-92a, miR-126, miR-143, miR-181b, and miR-221, increased vascular SMCs migration *in vitro* and inhibited that of ECs when compared with EVs from control patients (Sorrentino et al., 2020). Further, these authors also described that small EVs induced M1 polarization marker expression in macrophages *in vitro*, regardless of the pathophysiological condition from which EVs were isolated, control or PAD (Sorrentino et al., 2020). Moreover, the study of the transcriptional content of EVs might lead to the identification of new diagnosis and prognosis biomarkers, and therapeutic targets in PAD. In this regard, we found an upregulation of the S100A9 transcript after RNA-Seq analysis of plasma EVs in PAD patients (Saenz-Pipaon et al., 2020). Interestingly, as previously described (Engelberger et al., 2015; Dann et al., 2018), the protein encoded by S100A9, in circulation forming the heterocomplex S100A8/A9 or calprotectin, was elevated in PAD patients compared with controls and associated with a higher risk of mortality or amputation in the follow-up (Saenz-Pipaon et al., 2020), suggesting the potential of studying EVs content to identify novel biomarkers in chronic vascular diseases.

Regarding the biological activity of EVs, several authors have investigated their function in experimental models of hind limb ischemia using as source blood, tissues or cell culture conditioned medium. As such, in a rat model of femoral ischemia, platelet EVs from atherosclerotic patients increased the adhesion capacity of circulating angiogenic cells to the ischemic muscles and increased neovascularization, which was prevented when the coculture of EVs and circulating angiogenic cells was pretreated with a RANTES neutralizing antibody (Ohtsuka et al., 2013). In mouse, the proangiogenic effect of bone marrow–mononuclear cells was increased when administered together with EVs isolated from ischemic muscles in a mechanisms dependent on gp91 (Leroyer et al., 2009), while T lymphocyte-derived EVs enriched in Sonic hedgehog protein improved muscle recovery in a mechanism dependent on nitric oxide production (Benameur et al., 2010). Moreover, EVs from ETS variant transcription factor 2 transfected fibroblasts, increased neovascularization (Van Pham et al., 2017). In addition, recent findings indicate that the beneficial effects of stem cells in skeletal muscle repair might be partially mediated by EVs. As such, several authors reported improved muscle recovery by the intramuscular delivery of EVs generated by stem cells of different types, including mesenchymal, adipocyte, CD34^+^ or urine-derived (Hu et al., 2015; Gangadaran et al., 2017; Mathiyalagan et al., 2017; Ju et al., 2018; Lopatina et al., 2018; Zhu et al., 2018; Figliolini et al., 2020; Zhang et al., 2021). Overall, EVs seem to participate in all steps of vascular and muscle remodelling in PAD.

### Extracellular Vesicles: Possible Diagnostic and Prognostic Biomarkers in Aortic Stenosis

The possible utilization of EVs as diagnostic and prognostic biomarkers in AS, although still scarley studied, has been also addressed by several authors. Diehl et al. reported increased levels of leukocyte, platelet and endothelial derived EVs in patients with severe AS compared to controls (Diehl et al., 2008). In addition, they found a positive correlation between platelet and endothelial EVs, and shear stress and blood leukocyte numbers, respectively, in AS patients (Diehl et al., 2008). Other authors described that subjects with severe AS and high coronary calcification score presented elevated levels of circulating endothelial and platelet derived EVs, and augmented EVs-associated thrombin activity, when compared with AS subjects with low coronary calcification score (Horn et al., 2016), suggesting the involvement of EVs in endothelial dysfunction, inflammation and valvular calcification.

Regarding the possible role of EVs as biomarkers for outcome assessment after valvular replacement, Marchini et al. found no differences in the concentration of total EVs, or in the levels of endothelial, platelet or leukocyte EVs early after surgery (5 days) compared to pre-procedure levels (Marchini et al., 2016). However, other authors reported a gradual decrease in endothelial EVs at longer time periods post-valvular replacement (1, 3 and 6 months) (Horn et al., 2015; Jung et al., 2017), accompanied by an improvement in the endothelial function (Horn et al., 2015) and an increase in platelet EVs (Jung et al., 2017). When it comes to small EVs or exosomes, their numbers acutely decrease after surgical valve replacement (24 h), going back to pre-operative levels 7 days and 3 months post-procedure, suggesting no alteration of small EVs release in response to valvular replacement (Weber et al., 2020). However, in a subgroup of patients, these authors described a positive correlation between increased levels of small EVs and patient-prosthesis mismatch parameters, and suggested the possible prognostic value of small EVs to estimate emerging patient-prosthesis mismatch and adverse outcomes in patients undergoing surgical aortic valve replacement (Weber et al., 2020). Locally, the *ex vivo* release of Annexin V + EVs to the conditioned medium was similar in ortic valves from patients suffering calcified AS of different origins, and no correlation between the released EVs and calcium content was observed (Kochtebane et al., 2013). Even if the summarized data points towards a role of EVs as biological effectors and biomarkers in AS, larger studies are needed to clarify their involment in the processes leading to AS initiation and progression.

### Biological Role of Extracellular Vesicles in Aortic Aneurysms

As mentioned above, blood-borne EVs are mainly derived from activated platelets (Burnier et al., 2009), although leukocytes, red blood cells and ECs can also participate (Nomura, 2017). In this respect, platelet-derived EVs are increased in plasma of AAA patients (Touat et al., 2006) and TAA (Touat et al., 2008). Following a proteomic approach, we described increased ficolin-3 levels in EVs isolated from the plasma of patients with AAA (Martinez-Pinna et al., 2014). We also showed that ficolin-3 levels were increased in EVs of platelets from healthy subjects incubated with ADP, as well as in EVs isolated from AAA thrombus-conditioned media (Fernandez-García et al., 2017). The number of particles was higher in activated platelets and pathological tissue compared with healthy aorta. In addition, by flow cytometry, we observed that staining for platelets, and also leukocytes, was increased in EVs obtained from thrombus, compared with those from healthy aorta. Finally, ficolin-3 levels in plasma were associated to both AAA presence and evolution, suggesting its potential role as a diagnostic and prognostic biomarker (Fernandez-García et al., 2017). In addition, the contribution of neutrophils (Folkesson et al., 2015) and macrophages (Wang et al., 2019) to the release of EVs in human AAA was previously demonstrated. Moreover, in a latter study, the authors demonstrated that inhibiting small EVs biogenesis decreased experimental AAA associated to a reduction in MMP-2 expression (Wang et al., 2019), describing a pathogenic role of macrophage-derived small EVs production in AAA. Regarding TAA, where inflammatory cells are not present, vascular SMCs were tested to contribute to EVs release. In this study (Jia et al., 2017), mechanical stretch induced EVs production by vascular SMCs, which depended on endoplasmic reticulum (ER) stress. This process led to EC dysfunction and contributed to TAA formation, while an ER stress inhibitor blocked EV production *in vitro* and TAA formation and rupture. In addition, Han et al. demonstrated an upregulation of miR-106a in small EVs from plasma and tissue-conditioned media of AAA patients (Han et al., 2020). Interestingly, the authors demonstrated a pathogenic role of miR-106, by favoring vascular SMCs apoptosis and ECM degradation. However, the cell type involved in miR-106 upregulation was not identified. Moreover, treatment with EVs from mesenchymal stromal cells decreased AAA development and macrophage activation in mice by inducing miR-147 (Spinosa et al., 2018). All these data suggest a potential contribution of EVs with a positive or negative effect on AAs mechanisms and progression that depends on the cells of origin and the stimuli triggering EVs release.

## Role of Extracellular Vesicles in Pathological Mechanisms Involved in Vascular Remodeling

### Extracellular Vesicles and Phenotype Switch of Resident Vascular and Valvular Cells

Cardiovascular remodeling is a main driver of CVD, where vascular SMCs and valvular ICs play a central role. Most vascular SMCs in the vessel wall display a contractile phenotype, which allows them to maintain vascular tone. However, under pathological vascular remodeling, it is well established that vascular SMCs loses their contractile phenotype to one resembling other cell types. Novel technologies including vascular SMCs lineage tracing, single cell (sc)RNAseq of mouse and human atherosclerotic vessels, and human genomics, demonstrate a multipotential fate of dedifferentiated vascular SMCs to cell types including foam cells, osteochondrocytes or myofibroblasts, among others (Wirka et al., 2019; Pan et al., 2020; Hu Z. et al., 2021). Moreover, numerous studies have demonstrated the ability of valvular ICs to undergo osteogenic trans-differentiation (Rajamannan et al., 2005; Osman et al., 2006; Chen et al., 2009; Monzack and Masters, 2011). Similarly, vascular SMCs phenotypic switch, as shown by loss of contractility markers and increases in matrix metalloproteinases, preceded aortic aneurysm in mice. Very recently, it has been demonstrated that aneurysm formation was driven by extensive reprogramming of contractile medial vascular SMCs to mesenchymal stem cell (MSC)- derived cell types including adipocytes, chondrocytes, osteoblasts, as well as macrophages that increased over time (Chen et al., 2020). The progressive accumulation of these cells provoked the loss of elastin fibers, intramural calcifications, massive lipid uptake and extensive inflammation. All this data supports the importance of vascular SMCs and valvular ICs phenotypic switch in pathological cardiovascular remodeling, although the exact impact of each phenotype is still under debate.

Regarding the potential contribution of EVs to vascular SMCs phenotypic switch, the incubation of human vascular SMCs with platelet EVs decreased the expression of contractile proteins, while inducing a synthetic proinflammatory phenotype resulting in increased IL-6 secretion and vascular SMCs migration and proliferation, through mechanisms involving CD40 and P-selectin interactions (Vajen et al., 2017). Under uremic conditions, EC-derived EVs stimulated vascular SMCs proliferation via TGF-beta (Ryu et al., 2019). Treatment with endothelial EVs reduced the proliferation and migration of vascular SMCs as well as lipid accumulation in vascular SMCs, while this beneficial effect was abolished or even reversed when treated with LPS-derived endothelial cell EVs (Xiang et al., 2021). ECs stimulated with oxysterol 7-ketocholesterol induced NLRP3 inflammasome activation and increased secretion of EVs that contain IL-1β. These EC-EVs rich in IL-1β promoted synthetic phenotype transition of co-cultured vascular SMCs, whereas EVs from unstimulated ECs had the opposite effects (Yuan et al., 2020). These last two recent results suggest a potential protective effect of EC-derived EVs on vascular SMCs, which seem to be impaired when produced under proinflammatory conditions, mimicking the pathological vascular wall.

### The Role of Extracellular Vesicles in Vascular Calcification

Calcification has been associated to several CVDs, including atherosclerosis, aortic valve stenosis and aortic aneurysm. Calcium arterial coronary (CAC) score is a marker of CV events (Detrano et al., 2008). Initially, aortic valve calcification was also associated with cardiovascular events, although the association was attenuated after CAC was taken into account (Owens et al., 2012). More recently, it has been demonstrated that aortic valve calcification is independently associated with all-cause and CV deaths in patients with low coronary atherosclerosis burden (Han et al., 2021). Similarly, calcification in either the thoracic or the abdominal territory, has also been suggested as a potential contributor to aneurysm progression and mortality (Buijs et al., 2013; Chowdhury et al., 2018). The association of calcification with CV events depends on the arterial territory affected (Allison et al., 2012), but this association also differs between microcalcifications (<50 µm) or macrocalcifications (O’Neill et al., 2015). Macrocalcification in carotid atherosclerotic lesions correlated with a transcriptional profile typical for stable plaques, with altered vascular SMCs phenotype and ECM composition, and repressed inflammation (Karlöf et al., 2019). In contrast, microcalcifications that are present in high-risk atheroma, predicts adverse cardiovascular events, and is associated with increased morbidity and mortality (McEvoy et al., 2010). More recently, a prospective observational study has demonstrated that 18F-FDG and 18F-NaF PET/CT performed in the superficial femoral artery before and 6 weeks after angioplasty identified patients who would develop restenosis within 12 months (Chowdhury et al., 2020). Finally, in the prospective SoFIA3 study, aneurysms with higher 18F-fluoride uptake had 2.5 times more rapid aneurysm expansion, and experienced three times more AAA repair or rupture, compared with patients with less aneurysmal microcalcification (Forsythe et al., 2018). The deleterious effect of microcalcification on AAA expansion was further demonstrated in the experimental model of AngII (angiotensin II)-induced AAA in mice (Li et al., 2020). In this study, vascular SMCs–specific ablation of Runx2 (runt-related transcription factor 2) abrogated microcalcification and inhibited AngII-induced AAA formation. Similarly, conditional depletion of Runx2 in valvular ICs and sinus wall cells of LDLr−/−ApoB100 mice (a model of calcified aortic valve disease) improved aortic valve function by decreasing aortic valve calcification (Dharmarajan et al., 2021). All these data highlights the importance of vascular SMCs and valvular ICs-induced calcification in cardiovascular remodeling.

Previous studies identified vascular SMCs or valvular ICs expressing markers of osteochondrocytes, along with decreased expression of endogenous inhibitors of calcification, in pathological arteries and aortic valve lesions (Boström et al., 1993; O’Brien et al., 1995; Mohler et al., 2001; Rajamannan et al., 2003; Tyson et al., 2003; Dubis et al., 2016). There are several potential inducers of vascular SMCs or valvular ICs to an osteocondrogenic phenotype in atherosclerotic plaques, valves and aneurysms, including apoptosis, oxidative stress, mitochondrial dysfunction and probably, inflammation (through paracrine factors secreted by inflammatory cells) (Dweck et al., 2012; Durham et al., 2018; P ; Petsophonsakul et al., 2019). These modified vascular SMCs and valvular ICs favor vascular calcification through the release of calcifying EVs, a subpopulation traditionally known as matrix vesicles (MVs) (Shanahan et al., 2011). Later studies demonstrated that MVs are exosomes and show that factors that can increase exosome release can promote vascular calcification in response to environmental calcium stress (Kapustin et al., 2015). Interestingly, this study demonstrated that the inhibition of exosome release by a sphingomyelin phosphodiesterase 3 inhibitor blocks calcification. In addition, macrophages could also release calcifying EVs in atherosclerotic plaques and aortic valves (New et al., 2013; Passos et al., 2020).

**FIGURE 3:**
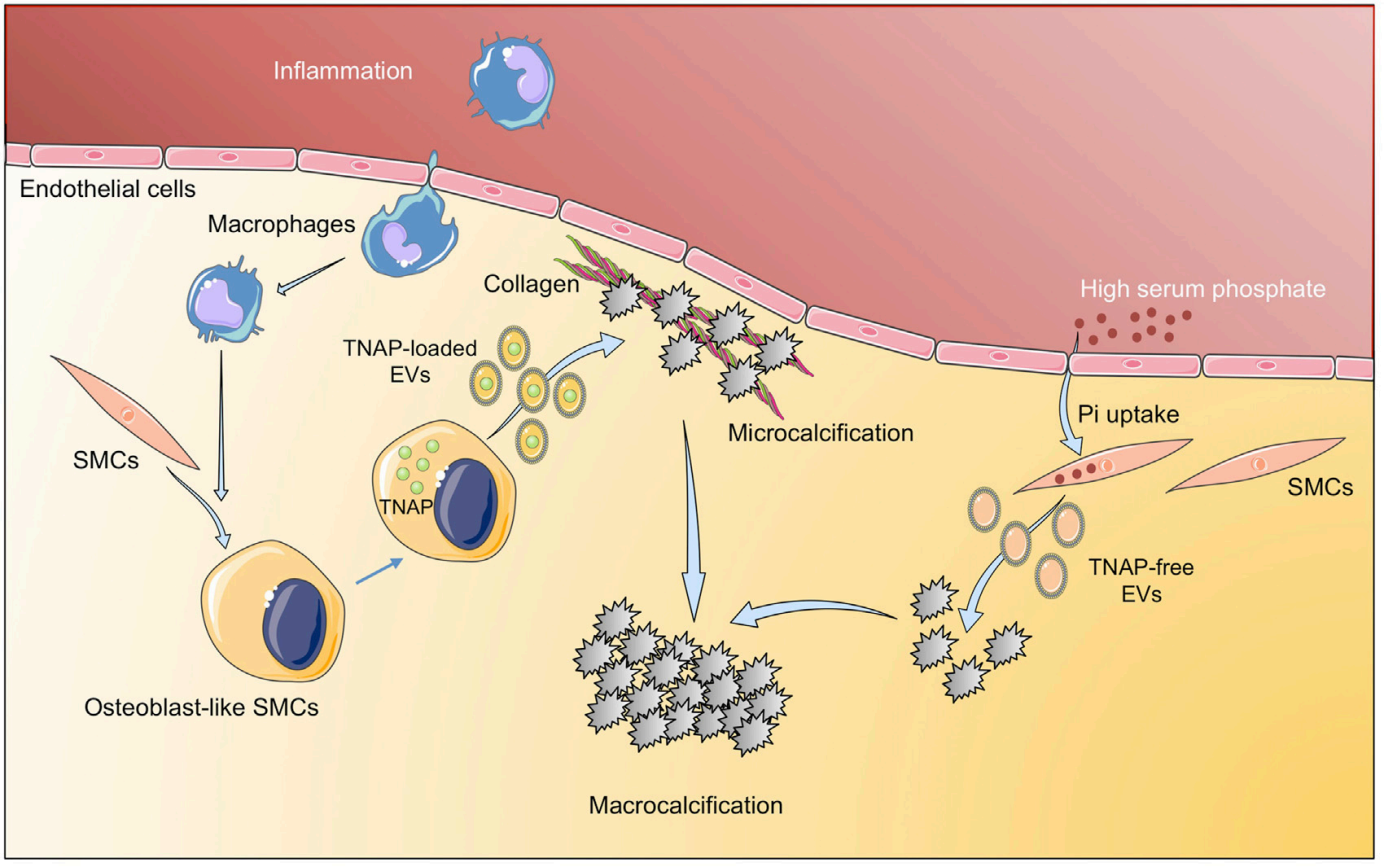
Implication of extracellular vesicles in vascular calcification. Inflammatory environment induces osteogenic differentiation of SMC to osteoblast-like SMCs that release TNAP-loaded EV that aggregate into microcalcifications. In addition, high serum phosphate induces tissue calcification through EV with low TNAP. Figure created using Servier Medical Art images (https://smart.servier.com).

Calcification, initially thought to be a passive process of calcium/phosphate precipitation in the ECMs, is nowadays described as an active-cell mediated process, which begins by aggregation of calcifying extracellular vesicles and the formation of microcalcifications, ultimate leading to large areas of calcification (Hutcheson et al., 2016). There are several events involved in vascular calcification, with a key role of EVs cargo as mediators of this process (Figure 3). The EVs cargo can be modified under a prolonged mineral imbalance and/or an inflammatory environment, which results in a reduction of calcification inhibitors inside EVs and their enrichment in protein–lipid complexes consisting of phosphatidylserine (PS) and Annexin A6 (Kapustin et al., 2011). Tissue nonspecific alkaline phosphatase (TNAP), a key enzyme during calcification through the hydrolysis of extracellular pyrophosphate into phosphate, has been also found in vascular SMCs- derived calcifying EVs (Chen et al., 2008). Interestingly, phosphate-induced calcification increased the expression and activity of TNAP in cultured vascular SMCs with a osteocondrogenic phenotype (Villa-Bellosta, 2018), and the overexpression of TNAP is sufficient to induce medial calcification in aortic rings *ex vivo* (Villa-Bellosta et al., 2011). During the calcification process, TNAP along with annexins, mediates the interaction of EVs released by vascular SMCs and valvular ICs with the ECMs (predominantly type I collagen), initiating the mineralization process (Côté et al., 2012; Mathieu et al., 2014). Thus, some studies have tried to interfere with the mediators/molecular pathways involved in the production and release of calcifying EVs, and/or with those participating in the switch of vascular SMCs to the chondrogenic phenotype. In this respect, one of the most studied mediators is discoidin domain receptor-1 (DDR-1), a collagen-binding tyrosine kinase receptor that regulates vascular calcification and atherosclerosis (Ahmad et al., 2009). Interestingly, DDR-1 (−/−) vascular SMCs exhibited a 4-fold increase in EV release with elevated TNAP activity (Krohn et al., 2016). Very recently, it has been demonstrated that DDR-1 regulates the transdifferentiation of vascular SMCs to osteochondrocytic cells by sensing matrix stiffening during disease progression and transmitting contractile forces through the actin cytoskeleton (Ngai et al., 2020). Another study highlighting the importance of EVs cargo described a novel mechanism involving sortilin, a member of the vacuolar protein sorting 10 protein family of sorting receptors, that has been related to calcification and CVDs (Goettsch et al., 2018). Interestingly, sortilin induced calcification by favoring the loading of activated TNAP into EVs, which was independent of vascular SMCs osteochondrogenic reprogramming and did not affect bone mineralization (Goettsch et al., 2016). Moreover, loss- and gain-of-function studies, did not reveal a sortilin-dependent change in the number and size of EVs from calcifying vascular SMCs, further supporting that sortilin-induced calcification is dependent on the modulation of EVs cargo rather than on EVs number or subpopulation. More recently, Annexin A1, a protein associated with calcium binding and intracellular endosomal transport, has been identified as a main component in EVs present in atherosclerotic plaques and has been involved in EV tethering, leading to aggregation and ectopic calcification (Rogers et al., 2020a). Similar to EVs derived from calcifying vascular SMCs, the pro-calcified valvular ICs derived EVs showed up-regulation of several annexins. An *in vitro* study demonstrated EVs secretion with elevated calcium and Annexin A6 from rat VICs cultured with high calcium and phosphate and suggested a role in calcified aortic valve disease evidenced by co-localization of Annexin A6 with EVs in the aortic valve (Cui et al., 2017). In addition to TNAP and annexins as mediators of cardiovascular calcification associated to their presence in EVs, other pathways have been studied. Two recent studies have analyzed the contribution of the Nox-5 subunit of NADPH oxidase in vascular calcification involving both, vascular SMCs phenotypic switch and EVs production. In a former study, it was demonstrated that switching from contractile to synthetic phenotype was required for vascular SMCs calcification, and that Ca^2+^-dependent Nox5 expression increased oxidative stress, leading to elevated vascular SMCs-EVs release and subsequent calcification (Furmanik et al., 2020). More recently, the same group described that Nox5 was also the underlying mechanism by which nicotine induced vascular SMCs calcification and EV secretion, further supporting the role of smoking in pathological vascular remodeling and calcification (Petsophonsakul et al., 2021).

In spite of all this progress on the knowledge of mechanisms involved in cardiovascular calcification, there are currently no approved drugs to prevent or treat calcification (Aikawa and Blaser, 2021). Regarding potential therapeutic strategies based on natural compounds, oligogalacturonic acid (present in smooth pectin regions of the apple cell wall matrix) reduced vascular calcification by inhibition of osteogenic phenotype of vascular SMCs, and also by preventing EVs binding to type I collagen (Hodroge et al., 2017). A recent study tested the role of retinoids on vascular calcification, showing that acyclic retinoid inhibited cardiovascular cell calcification by attenuating TNAP activity and Runx2 expression without adverse effects on bone mineralization (Rogers et al., 2020b). However, these studies need to be performed *in vivo* to test its potential application. In this respect, a selective and orally bioavailable TNAP inhibitor attenuated the development of calcification in mice *in vivo*, without the deleterious effects on bone associated with other proposed treatment strategies (Ziegler et al., 2017; Tani et al., 2020); however, whether these strategies involved EVs and whether they could be applied in models of pathological cardiovascular remodeling deserve further studies.

## Conclusion

Circulating EVs have been postulated as potential biomarkers in CVDs, as their absolute numbers, or the number of specific subpopulations have been associated to the incidence and prognosis of CVDs. In order to find novel biomarkers, different approaches on EVs have been used, from analysis of vascular SMCs-derived EVs under mineralization conditions (Kapustin et al., 2011), to AAA-tissue-derived EVs (Fernandez-García et al., 2017; Han et al., 2020) or plasma EVs from atherosclerotic patients (Madrigal-Matute et al., 2014; Saenz-Pipaon et al., 2020), among others. However, while many of these biomarkers demonstrate prognostic associations with CVD clinical outcomes, future research will be required to clarify their mechanistic roles and their potential clinical utility. Furthermore, current advances in -Omic approaches, mainly focused on improving sensitivity, will be crucial to dissect the molecular content of EVs in different pathological conditions, leading to the identification of differentially expressed RNAs, protein or metabolites, that will help to delineate the molecular profiles of different CVDs, and to identify novel diagnosis and prognosis biomarkers, as well as therapeutic targets.

In addition, EVs-based therapeutics have been proposed for several pathologies, including CVDs (Herrmann et al., 2021). Recently, the coating of stents with EVs resulted in accelerated re-endothelization and reduced instent re-stenosis compared to drug-eluting or bare-metal stents in mice (Hu S. et al., 2021). Similarly, the administration of EVs secreted by immortalized cardiosphere-derived cells, engineered to express high levels of b-catenin, modulated the immune response and improved cardiac function in an experimental model of cardiomyophaty in mice (Lin et al., 2021). We can thus envision the possibility to engineer EVs to modify their cargo and prevent vascular calcification and pathological remodeling. In this respect, Ldlr mRNA was encapsulated into EVs and then injected in atherosclerotic LDLR null mice, decreasing both hepatic steatosis and atherosclerotic lesions (Li et al., 2021). The translation of these experimental studies to humans is awaiting, and several clinical trials are in progress (Sahoo et al., 2021). At present we are only aware of one study performed with peripheral blood mononuclear cells obtained from patients with anthracycline-induced cardiomyopathy, reprogrammed into induced pluripotent stem cells and differentiated into cardiomyocytes. These cardiomyocytes were treated with EVs from mesenchymal stromal cells resulting in preserved mitochondrial function, augmentation of ATP production, mitigation of ROS production, and suppression of apoptosis (O’Brien et al., 2021), being all these processes involved in vascular calcification and pathological remodeling. In this study, large but not small EVs, had a therapeutic activity, while other studies in different pathologies have suggested the opposite view (Tieu et al., 2021), thus highlighting the need for a better standardization and characterization of the effect of different EVs subpopulations for translational therapeutic purposes.
